# Draft Genome Sequence of Hydrocarbon-Degrading *Staphylococcus saprophyticus* Strain CNV2, Isolated from Crude Oil-Contaminated Soil from the Noonmati Oil Refinery, Guwahati, Assam, India

**DOI:** 10.1128/genomeA.00370-16

**Published:** 2016-05-12

**Authors:** Arghya Mukherjee, Bobby Chettri, James S. Langpoklakpam, Arvind K. Singh, Dhrubajyoti Chattopadhyay

**Affiliations:** a Department of Biotechnology, University of Calcutta, Kolkata, West Bengal, India; b Department of Biochemistry, North-Eastern Hill University, Shillong, Meghalaya, India

## Abstract

Here, we report the 2.6 Mb draft genome sequence of hydrocarbon-degrading *Staphylococcus saprophyticus* strain CNV2, isolated from oil-contaminated soil in Guwahati, India. CNV2 contains 2,545 coding sequences and has a G+C content of 33.2%. This is the first report of the genome sequence of an *S. saprophyticus* adapted to an oil-contaminated environment.

## GENOME ANNOUNCEMENT

*Staphylococcus saprophyticus* is a Gram-negative, coagulase-negative cocci, commonly associated with urinary tract infections (1). Until now, very few studies have reported hydrocarbon-degrading attributes in an *S. saprophyticus* strain (2). However, to date no genome sequence has been available for *S. saprophyticus* inhabiting oil-contaminated environments. In the present study, we report sequencing of the entire genome of *S. saprophyticus* strain CNV2, isolated from crude oil-contaminated soil collected from the Noonmati oil refinery in Guwahati, Assam, India. Strain CNV2 was found to efficiently degrade n-hexane, n-hexadecane, diesel oil, and crude oil. Hence, the genome of this organism was sequenced to obtain better insights into the metabolic versatility and adaptability of this strain. To our knowledge, this is the first report of the genome sequence of an *S. saprophyticus* strain adapted to an oil-contaminated environment.

The genome of strain CNV2 was extracted using using an UltraClean Microbial DNA Isolation Kit (MoBio Laboratories, Carlsbad, CA, USA) according to the manufacturer’s guidelines. The purified genome was then sequenced using an Illumina HiSeq 2500 platform, generating 2,423,395 high-quality paired-end reads. Quality filtered reads were then assembled using *de novo* assemblers ABySS v3.81 (3), Edena v3.130110 (4), MaSuRCA v2.2.1 (5), SOAPdenovo2 v2.04 (6), SPAdes v3.1.1 (7), and Velvet v1.2.10 (8). Integration of the assembled contigs was carried out using CISA v1.3 (9) resulting in five contigs with a *N*_50_ length of 2,344,483 bp and an average length of 527,180 bp. The draft genome sequence was 2,635,899 bp in length, with a G+C content of 33.2% and 65-fold coverage. Genome annotation was carried out in the NCBI Prokaryotic Genome Annotation Pipeline which predicted the presence of 2,484 protein coding sequences (CDSs), 29 pseudogenes, 9 rRNAs, 42 tRNAs, 4 noncoding RNAs (ncRNAs), and a clustered regularly interspaced short palindromic repeat (CRISPR) array. Rapid genome annotation for CNV2 carried out in the RAST annotation server (10) classified predicted CDSs into 398 subsystems, among which cofactors, vitamins, prosthetic groups and pigments (*s* = 159 CDSs), cell wall and capsule (*s* = 108), virulence, disease and defense (*s* = 51), RNA metabolism (*s* = 125), nucleosides and nucleotides (*s* = 88), protein metabolism (*s* = 207), DNA metabolism (*s* = 77), fatty acids, lipids, and isoprenoids (*s* = 117), stress response (*s* = 75), amino acids and derivatives (*s* = 331), and carbohydrates (*s* = 290) were the most abundant ones. The presence of an alkane-1-monoxygenase gene and large genetic investments in heavy metal and antibiotic resistance genes along with stress response genes indicate that CNV2 is highly adapted to different stress conditions, including oil contamination.

A comparison of strain CNV2 with genomes in the RAST database identified *S. saprophyticus* ATCC 15305 (score = 507) as its closest neighbor, followed by *S. equorum* strain Mu2 (score = 447) and *S. saprophyticus* KACC 16562 (score = 433). *S. epidermidis* M23864:W1 (score = 354) was identified as the eighth closest neighbor.

### Nucleotide sequence accession numbers.

This whole-genome shotgun sequencing project for *S. saprophyticus* strain CNV2 has been deposited in DDBJ/EMBL/GenBank under the accession no. LUGM00000000. The version of the whole-genome sequence (WGS) described here is version LUGM01000000.
